# Operational tactical uniforms promote body schema underestimation and musculoskeletal discomfort in Brazilian male special operations police officers

**DOI:** 10.3389/fpubh.2026.1899955

**Published:** 2026-09-04

**Authors:** Roberta L. Rica, Eliane F. Gama, Geanderson S. Oliveira, Matheus F. de Deus, Michell V. Viana, Pedro F. C. Fortes Junior, Valentina Bullo, Marco Bergamin, Stefano Gobbo, Danilo S. Bocalini

**Affiliations:** 1 Department of Physical Education, Estácio de Sá University Center, Vitoria, Espírito Santo, Brazil; 2 Department of Morphology and Genetics, Federal University of São Paulo, São Paulo Medical School, São Paulo, São Paulo, Brazil; 3 Experimental Physiology and Biochemistry Laboratory, Department of Morphology and Genetics, Federal University of Espírito Santo, Vitoria, Espírito Santo, Brazil; 4 Department of Medicine, University of Padova, Padova, Italy; 5 Gymhub S.r.l. Spin-off of the University of Padova, Padova, Italy

**Keywords:** body perception, ergonomics, musculoskeletal disorders, personal protective equipment, police officers

## Abstract

**Background:**

Tactical police officers are routinely exposed to high occupational demands and external load carriage resulting from operational uniforms and personal protective equipment (PPE). Although PPE is essential for operational safety, its effects on body schema perception and musculoskeletal discomfort remain poorly understood in elite police populations.

**Objective:**

To investigate the effects of operational uniforms and PPE on body schema perception and musculoskeletal discomfort in special operations police officers.

**Methods:**

Fifteen police officers were evaluated under two experimental conditions, physical training uniform (PTU) and operational uniform with PPE (OU). Musculoskeletal discomfort was assessed using the Corlett and Manenica Body Map, while body schema perception was evaluated using the Image Marking Procedure and Body Perception Index (BPI).

**Results:**

military officers age was 37.57 ± 6.08 years, time service of 13.33 ± 5.99 years and elevated body mass index 28.49 ± 3.60 kg/m^2^. An additional load of 14.79 ± 1.83 kg Operational uniforms increased external body load and significantly elevated musculoskeletal discomfort scores across most body regions (*p* < 0.05). In addition, body schema perception significantly decreased under the operational condition, demonstrating body dimension underestimation and predominance of hypo-schematism.

**Conclusion:**

Operational uniforms and PPE promoted increased musculoskeletal discomfort and altered body schema perception in tactical police officers, potentially affecting occupational safety and operational performance.

## Introduction

Recent evidence has reinforced the high prevalence of musculoskeletal disorders among police officers exposed to prolonged operational load and tactical demands. A recent epidemiological investigation involving officers from a German federal state police force demonstrated elevated prevalence of musculoskeletal complaints, particularly in the lumbar spine, neck, shoulders, and lower limbs, highlighting the substantial occupational burden associated with police activities (1). According to the authors, prolonged use of operational equipment, repetitive tactical movements, static postures, and occupational stress were identified as major contributors to musculoskeletal overload and reduced occupational wellbeing. These findings corroborate previous studies demonstrating that tactical equipment and occupational load carriage substantially increase biomechanical stress, fatigue, and discomfort in law enforcement personnel (2, 3).

Among tactical police officers, the use of personal protective equipment (PPE) and operational gear is mandatory to ensure safety during all missions. Tactical equipment generally includes ballistic vests, tactical belts, firearms, ammunition, radios, helmets, handcuffs, communication devices, and boots, substantially increasing external body load (4). Depending on operational demands, police officers may carry loads ranging from approximately 10–40 kg, resulting in increased physiological stress, fatigue, movement restriction, and biomechanical overload (2, 3, 5). Previous military investigations demonstrated that external load carriage negatively affects obstacle negotiation, locomotor efficiency, and tactical task performance, particularly under conditions requiring rapid displacement and spatial adjustment (6).

Marins et al. (3) had demonstrated a high prevalence of musculoskeletal symptoms among police officers, especially in the lumbar spine, shoulders, neck, knees, and dorsal regions. Furthermore, the use of tactical operational uniforms has been associated with increased musculoskeletal discomfort, impaired mobility, altered gait mechanics, and reduced physical performance (7–9). Recent evidence involving Brazilian special operations police officers also demonstrated that operational uniforms significantly increase musculoskeletal discomfort and biomechanical overload during tactical tasks (2).

Systematic investigation (10) involving police populations have also demonstrated that occupational exposure to tactical equipment, prolonged standing, repetitive movements, and operational stress may substantially compromise physical health and cardiometabolic parameters in law enforcement professionals. Furthermore, musculoskeletal injuries associated with operational equipment use may negatively affect occupational functionality and quality of life among police officers.

From a neurophysiological perspective, prolonged exposure to tactical load carriage may overload proprioceptive pathways and alter cortical processing associated with body representation and movement planning (11). Recent studies (12, 13) have also emphasized that restrictive occupational equipment may reduce kinesthetic awareness and impair the continuous updating of body schema necessary for accurate interaction with the surrounding environment. These mechanisms may partially explain the perceptual compression observed in the present investigation.

Body schema perception is a fundamental component of sensorimotor control, representing the dynamic internal representation of body position, dimensions, and movement derived from the continuous integration of visual, proprioceptive, and tactile sensory information. This neural representation enables individuals to accurately plan and execute motor actions while adapting to changes in body configuration or environmental constraints. Contemporary neuroscience studies suggest that external objects, tools, and wearable equipment may temporarily modify body schema representation and peripersonal space perception (11, 12). Although adaptive plasticity allows the incorporation of tools into body representation, excessive load carriage and restrictive operational equipment may impair proprioceptive accuracy and sensorimotor integration (4, 14). These alterations may compromise tactical movement precision, obstacle negotiation, spatial judgment, and operational safety.

Alterations in body schema may impair movement efficiency, spatial awareness, postural control, and interaction with external objects, potentially compromising operational performance and increasing the risk of errors or injury. In occupations requiring high levels of precision and rapid decision-making, such as military and law enforcement operations, understanding how external factors, including personal protective equipment (PPE) influence body schema perception is essential for optimizing performance, safety, and ergonomic interventions. However, despite increasing scientific interest in occupational load carriage, the relationship between operational police uniforms and body schema perception remains poorly explored in elite police populations.

This gap is particularly relevant because underestimation of body dimensions while wearing PPE may expose officers to operational risks during confined-space movement, tactical entries, and cover utilization. Inaccurate perception of body dimensions may impair concealment strategies, spatial decision-making, and movement efficiency during critical incidents (15). Therefore, understanding how operational equipment influences body schema perception may provide important insights for occupational ergonomics, injury prevention, and tactical performance optimization. Thus, the present study aimed to analyze the effects of operational uniforms and PPE on body schema perception and musculoskeletal discomfort in military police officers from the Special Operations Company of Espírito Santo, Brazil.

## Materials and methods

### General characteristics and sample selection

The study design corresponding to within-subject, two-condition (PTU vs. OU) quasi-experimental comparison. Thus, after approval by the Human Research Ethics Committee of the Federal University of Espírito Santo (protocol number 6,275,609/2023), male military police officers from the Special Operations Company (SOC) of the Military Police of the State of Espírito Santo were invited to voluntarily participate in the study. The unit is allocated within the Special Missions Battalion (SMB), headquartered in the Jardim América district, municipality of Cariacica, Espírito Santo, Brazil.

All participants who agreed to take part in the study signed an Informed Consent Form, in accordance with the guidelines established by Resolution No. 466/2012 of the Brazilian National Health Council. Recruitment was conducted through direct contact with the officers, as well as through verbal and digital dissemination strategies. Military police officers, actively engaged in occupational duties, were included in the study. Non-inclusion criteria comprised military personnel on leave for any reason during the study period, as well as failure to sign the informed consent form. Participants who did not complete all stages of data collection and analysis were excluded. After applying inclusion and exclusion criteria, the final sample consisted of 45 officers from the SOC participated in this study, representing, 50% of specific population.

### Data collection procedures

The SMB comprises elite units of the Military Police of Espírito Santo that employ specialized tactics and techniques in high-complexity operations, including civil disturbance control, incidents involving explosive devices, and hostage situations. The battalion currently consists of approximately 200 officers and is considered the last-resort unit of the Military Police, characterized by advanced technical training, continuous preparation, loyalty, and strong commitment to duty. In general, SOC officers are trained in operations involving explosive devices, chemical agents, high-angle rescue, aquatic operations, prison interventions, tactical entries, crisis management, hostage negotiation, and precision shooting.

Data collection followed procedures similar to previous studies conducted by our research group (2) and was carried out in the workplace during duty days, according to participants' availability by experienced researcher in all procedures. After a full explanation of the study, participants completed the questionnaires and received detailed instructions regarding the procedures. The sequence of assessments was as follows: (1) reading and signing the informed consent form, (2) musculoskeletal discomfort assessment by paper-pencil format, (3) anthropometric evaluation using physical training uniform (PTU) and operational uniform (OU), and (4) randomized assessment of body schema perception according to PTU or OU use.

### Evaluated parameters

#### Anthropometric measures

Body mass was measured using a digital scale (MarteScientific, LS200P) with a precision of 0.1 kg, and height was measured using a stadiometer (Cardiomed, model WCS) with a precision of 0.1 cm. Body mass index (BMI) was calculated as body mass divided by height squared. The BMI values (kg/m^2^) were classified as: normal weight (18.5–24.9), overweight (25.0–29.9), and obesity (30.0–39.9).

#### Musculoskeletal discomfort

Musculoskeletal discomfort was assessed using the Corlett and Manenica Body Map (1980), previously applied in studies conducted by our group (2). This instrument evaluates both the location and intensity of pain by dividing the body into 27 segments, with a pain scale ranging from 1 (no pain) to 5 (extreme pain).

A total discomfort score was calculated by summing the scores across all 27 body segments. Perceived musculoskeletal discomfort was assessed under two conditions: without personal protective equipment (PPE) using PTU and OU with PPE. The operational uniform includes mandatory equipment such as handcuffs, radios, ballistic vest, firearms and ammunition, boots, and communication devices, totaling approximately 15 kg.

#### Body dimension schema

Body dimension schema was assessed using the Image Marking Procedure, as previously described (16). Briefly, tactile stimulation was applied to predefined anatomical landmarks, and all participants were marked with adhesive tape at the right and left acromioclavicular joints, waist curves, and greater trochanters using colored markers to ensure consistency across trials.

Participants stood near a wall with a vertically fixed measuring tape serving as a reference. Upon tactile stimulation of each marked point, participants were asked to indicate the orthogonal projection of that point on the wall. Each indicated point was marked with a colored label. The sequence began with the top of the head, followed by the right shoulder, waist, and hip, then the left side.

The test was repeated three times, and participants were not allowed to see previous markings. After recording perceived measurements, participants (blindfolded) were positioned close to the wall to obtain actual body measurements using the measuring tape. All markings were photographed and later analyzed using AxioVision software (Version 3.1).

Quantitative analyses were performed by calculating distances between perceived and actual points, evaluating both height and width in the horizontal plane. Data were tabulated, and the body perception index was calculated as the ratio between perceived and actual measurements multiplied by 100. The total BPI was computed as the average of regional BPIs (16). Values above 112.3% indicated hyper-schematism, whereas values below 99.4% indicated hypo-schematism.

#### Statistical analysis

Data are presented as absolute (*n*) and relative (%) frequencies for categorical variables, and as mean ± standard deviation, coefficient of variation (CV), and 95% confidence interval (95% CI) for quantitative variables. Normality was assessed using the D'Agostino–Pearson test. Comparisons between conditions (with and without PPE) were performed using the chi-square test for categorical variables (body schema perception classification) and the paired t-test for continuous variables. Effect size was estimated using Hedges' g and interpreted as small (0.2–0.5), moderate (0.5–0.8), or large (>0.8). Additionally, Pearson's correlation test was used to assess the strength and direction of associations between musculoskeletal discomfort scores and BPI values. Correlation strength was classified as weak (*r* = 0.1–0.3), moderate (*r* = 0.4–0.6), and strong (*r* = 0.7–1.0). Statistical analyses were performed using GraphPad Prism version 6.00 for Windows (GraphPad Software, La Jolla, CA, USA), with a significance level set at p < 0.05.

## Results

As shown in Table 1, military officers age was 37.57 ± 6.08 years, with a mean service time of 13.33 ± 5.99 years. Overall, the sample presented elevated body mass index values, and an additional load of 14.79 ± 1.83 kg (coefficient of variation: 12.42%) corresponding to personal protective equipment incorporated into the operational uniform.

**Table 1:** General characteristics.

Parameters	Mean ±SD	CV (%)	95% CI
Age (years)	37.57 ± 6.08	16.20	34.80–40.34
Service time (years)	13.33 ± 5.99	44.96	10.60–16.06
Body mass PTU (kg)	85.55 ± 11.03	12.90	80.53–90.57
Body mass OU (kg)	100.33 ± 10.70	10.66	95.47–105.20
Mass of PPE (kg)	14.79 ± 1.83	12.42	13.95–15.63
Height (m)	1.73 ± 0.07	4.43	1.69–1.76
BMI (kg/m^2^)	28.49 ± 3.60	12.64	26.85–30.13

Values are expressed as mean ± standard deviation, coefficient of variation (CV), and 95% confidence interval (95% CI). PTU, physical training uniform; OU, operational uniform; PPE, personal protective equipment.

Table 2 presents the results related to the perception of musculoskeletal discomfort among the military personnel. No significant differences were found (*p* > 0.05) between the right and left sides when considering the overall assessment. However, only the left arm and forearm, both wrists, and the right hand showed no significant differences when comparing the conditions with PTU and OU.

**Table 2:** Perception of musculoskeletal discomfort among military police officers with and without personal protective equipment.

Parameters		PTU	OU	95% CI	ES	*p*
Neck		1.38 ± 0.86	2.09 ± 1.09	0.23–1.19	0.70 (moderate)	0.0056
		(61.61%)	(52.07%)			
Upper back		1.33 ± 0.73	2.00 ± 1.14	0.27–1.05	0.68 (moderate)	0.0019
		(54.77%)	(57.01%)			
Middle back		1.38 ± 0.86	2.14 ± 1.23	0.35–1.16	0.70 (moderate)	0.0008
		(62.61%)	(57.70%)			
Lower back		1.76 ± 1.17	2.71 ± 1.23	0.42–1.48	0.77 (moderate)	0.0012
		(66.93%)	(45.34%)			
Pelvis		1.23 ± 0.76	2.00 ± 1.30	0.24–1.27	0.70 (moderate)	0.0060
		(67.07%)	(65.19%)			
Shoulders	Right side	1.28 ± 0.78	1.95 ± 1.20	0.20–1.12	0.64 (moderate)	0.0070
		(60.96%)	(61.63%)			
	Left side	1.38 ± 0.97	1.90 ± 1.04	0.03–1.01	0.50 (moderate)	0.0376
		(70.49%)	(54.82%)			
Arms	Right side	1.14 ± 0.65	1.76 ± 1.22	0.11–1.12	0.62 (moderate)	0.0195
		(57.28%)	(69.29%)			
	Left side	1.33 ± 1.06	1.66 ± 1.06	−0.12 to 0.79	0.30 (small)	0.1485
		(79.84%)	(63.87%)			
Forearms	Right side	1.14 ± 0.78	1.47 ± 0.98	−0.12 to 0.79	0.37 (small)	0.1485
		(57.28%)	(66.44%)			
	Left side	1.19 ± 0.87	1.38 ± 0.74	−0.17 to 0.56	0.23 (small)	0.2961
		(73.32%)	(53.59%)			
Wrists	Right side	1.23 ± 0.76	1.66 ± 1.15	−0.01 to 0.87	0.43 (small)	0.0584
		(62.07%)	(69.28%)			
	Left side	1.23 ± 0.76	1.61 ± 1.07	−0.01 to 0.77	0.40 (small)	0.0571
		(62.07%)	(66.17%)			
Hands	Right side	1.09 ± 0.43	1.33 ± 0.65	−0.00 to 0.48	0.42 (small)	0.0565
		(39.85%)	(49.37%)			
	Left side	1.09 ± 0.43	1.47 ± 0.81	0.04–0.71	0.57 (moderate)	0.0286
		(39.85%)	(55.11%)			
Thighs	Right side	1.04 ± 0.21	1.66 ± 0.96	0.17–1.06	0.87 (large)	0.0086
		(20.83%)	(57.97%)			
	Left side	1.04 ± 0.21	1.76 ± 0.99	0.25–1.17	0.98 (large)	0.0040
		(20.83%)	(54.49%)			
Legs	Right side	1.42 ± 0.97	2.00 ± 1.09	0.17–0.96	0.55 (moderate)	0.0069
		(68.48%)	(54.77%)			
	Left side	1.57 ± 1.12	1.95 ± 1.24	−0.03 to 0.80	0.31 (small)	0.0725
		(71.35%)	(63.77%)			
Ankles and feet	Right side	1.23 ± 0.76	2.04 ± 1.16	0.31–1.30	0.80 (large)	0.0026
		(62.07%)	(56.69%)			
	Left side	1.28 ± 0.90	2.09 ± 1.26	0.29–1.32	0.72 (moderate)	0.0036
		(70.18%)	(60.19%)			
Overall discomfort		26.71 ± 9.32	38.71 ± 15.26	5.70–18.30	0.92 (large)	0.0007
		(34.90%)	(39.42%)			

Values are expressed as mean ± standard deviation, coefficient of variation (CV), and 95% confidence interval (95% CI). PTU, physical training uniform; OU, operational uniform; PPE, personal protective equipment.

**Figure 1:**
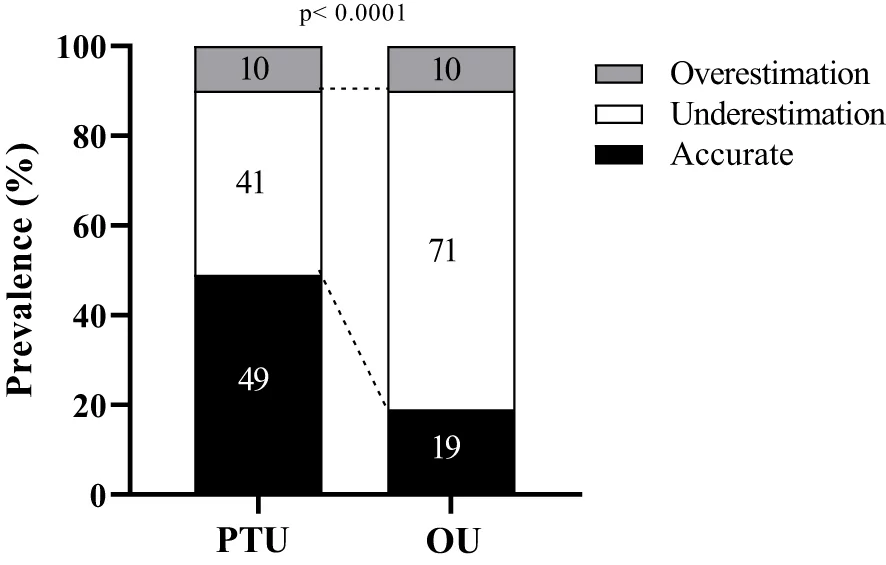
Prevalence of body schema perception classification among Special Operations Company officers under physical training uniform (PTU) and operational uniform (OU) conditions.

Regarding body schema perception, as shown in Table 3, values ranged from the lowest in the shoulder BPI segment (92.64 ± 15.10; CV: 16.30%) to among the highest values in the hip and waist regions under the PTU condition. Under the OU condition, the lowest value was also observed in the shoulder segment (82.35 ± 15.70; CV: 19.06%), while higher values remained concentrated in the hip and waist regions, indicating reduced perception across all body segments when wearing the operational uniform. As showed at Figure 1, differences were found of body schema perception between PTU and OU conditions.

**Table 3:** Body schema perception of military police officers with and without personal protective equipment.

Parameters	PTU	OU	95% CI	ES	*P*
General BPI	100.80 ± 13.57	92.63 ± 12.74	−13.38 to −2.99	0.60 (moderate)	0.003
	(13.46%)	(13.75%)			
Heights	93.41 ± 6.01	89.86 ± 4.07	−6.89 to −0.22	0.67 (moderate)	0.037
	(6.43%)	(4.53%)			
Shoulder	92.64 ± 15.10	82.35 ± 15.70	−15.39 to −5.19	0.65 (moderate)	0.001
	(16.30%)	(19.06%)			
Hip	110.20 ± 20.40	97.12 ± 19.40	−19.68 to −6.57	0.64 (moderate)	0.001
	(18.50%)	(19.97%)			
Waist	107.30 ± 21.64	97.11 ± 16.92	−18.05 to −2.31	0.51 (moderate)	0.013
	(20.17%)	(17.42%)			

Values are expressed as mean ± standard deviation, coefficient of variation (CV), and 95% confidence interval (95% CI). PTU, Physical training uniform; OU, operational uniform; BPI, body schema perception index

**Figure 2:**
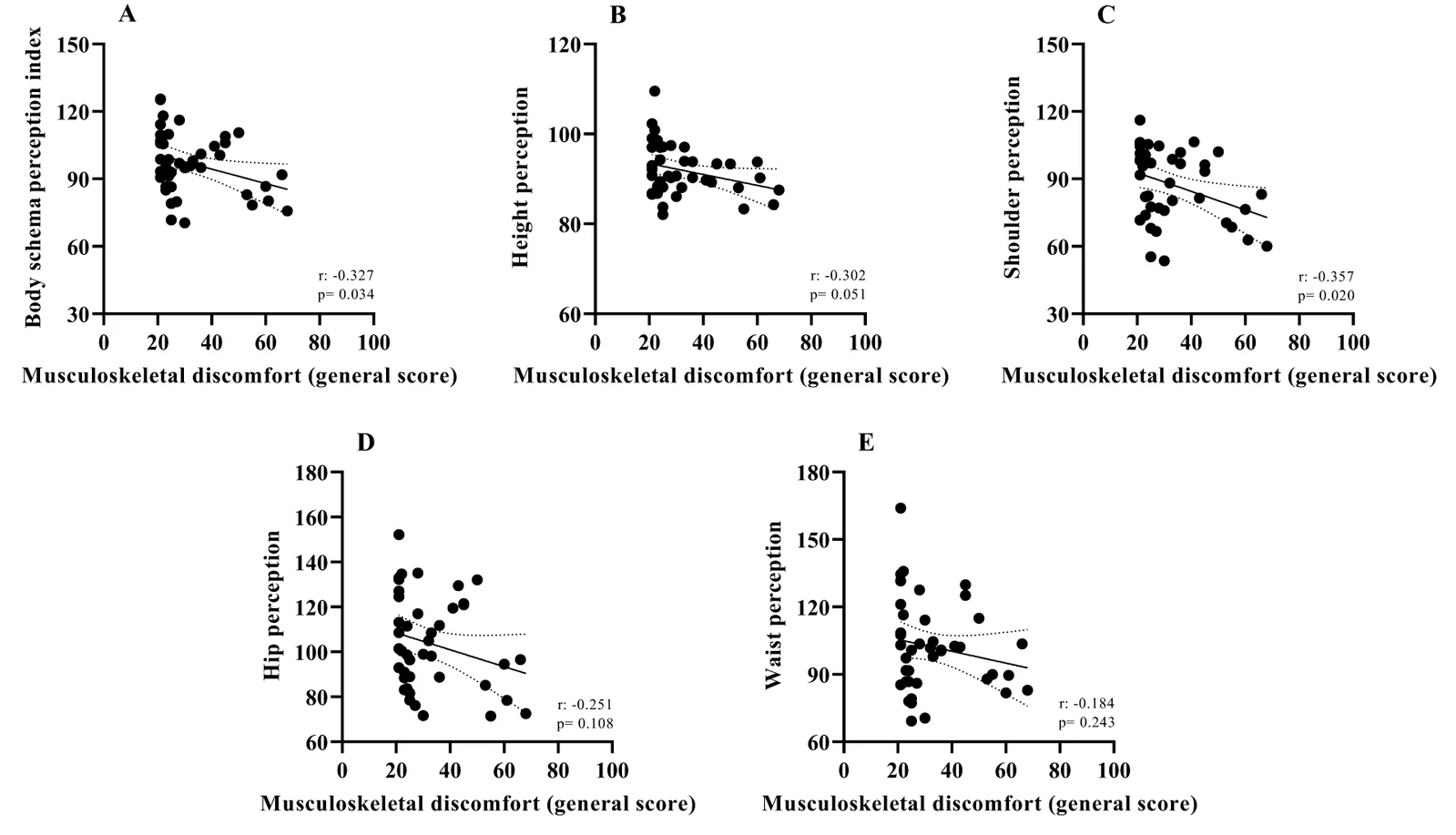
Correlations between values of body schema perception **(A)**, height **(B)**, shoulder **(C)**, hip **(D)**, waist **(E)** parameters and overall musculoskeletal discomfort in SOC officers. **(A)** Body scheme perception index.

On Figure 2 are presents the correlations between body schema perception values and overall musculoskeletal discomfort among SOC officers. Although negative correlation was observed just in shoulder segment (*r* = −0.357, *p* = 0.020) and the body schema perception index (*r* = −0.327, *p* = 0.034). No significant correlations (*p* > 0.05) were identified between another body schema parameters values and overall musculoskeletal discomfort.

## Discussion

Police officers from the Special Operations Company (SOC) of the Special Missions Battalion (SMB) of Espírito Santo, Brazil, perform highly specialized tactical activities requiring elevated physical preparedness, rapid motor responses, and advanced operational capability. These occupational demands are directly associated with physical fitness, musculoskeletal integrity, and perceptual-motor efficiency necessary to ensure tactical performance and officer safety (5). Accordingly, the present study investigated the effects of operational uniforms and personal protective equipment (PPE) on musculoskeletal discomfort and body dimension perception in this elite tactical population.

Operational police officers routinely wear protective equipment that may add between 10 and 55 kg of external load depending on mission characteristics and tactical specialization (2, 4). Although essential for ballistic and operational protection, recent studies have demonstrated that tactical load carriage substantially increases biomechanical stress, movement restriction, physiological demand, fatigue, and musculoskeletal overload (3, 7, 13). More recent investigations involving tactical populations have also demonstrated that prolonged exposure to operational equipment significantly impairs occupational ergonomics, increases lumbar overload, reduces mobility, and compromises dynamic balance, movement precision, and postural control during high-intensity operational tasks and prolonged tactical exposure (1, 2, 18).

In the present study, the operational uniform increased body mass by approximately 14.79 kg due to the additional load imposed by PPE. This finding is consistent with previous investigations involving military and police personnel demonstrating that ballistic vests, tactical belts, communication systems, and firearms negatively affect locomotion efficiency and increase physiological stress during tactical tasks (9, 13, 17). Additionally, prolonged load carriage may alter gait mechanics, postural stability, and neuromuscular activation patterns, increasing susceptibility to chronic musculoskeletal disorders (4, 18).

Musculoskeletal discomfort significantly increased across most evaluated body regions under operational conditions. The greatest discomfort scores were observed in the lumbar spine, shoulders, and lower limbs, corroborating previous studies demonstrating elevated prevalence of musculoskeletal symptoms among police officers exposed to prolonged operational load and vehicle patrol (1, 3, 19, 20). Recent tactical ergonomics investigations also demonstrated that ballistic equipment and operational load distribution substantially increase spinal compression forces and postural compensations in elite police officers (2).

A central finding of the present study was the significant reduction in Body Perception Index (BPI) under operational conditions, indicating underestimation of body dimensions. This perceptual alteration was particularly pronounced in the shoulder region, which is critical for spatial negotiation, tactical displacement, and cover utilization. Similar findings have been reported in studies involving perceptual distortion and body awareness in adult men (14, 15). These investigations suggest that altered proprioceptive feedback and elevated attentional demand may contribute to inaccurate perception of body dimensions.

Recent neuroscience evidence (11, 12) indicates that wearable equipment and external objects may temporarily modify spatial perception and motor adaptation mechanisms. However, the present findings suggest that the additional tactical load was not fully incorporated into participants' perception of their own body dimensions. Instead of adapting to the increased external volume produced by PPE, officers tended to underestimate their actual occupied space while wearing operational equipment.

This phenomenon may be partially explained by the mechanical and physiological effects of tactical load carriage. Previous military ergonomics studies demonstrated that external load promotes localized fatigue, altered gait kinematics, and reduced proprioceptive accuracy due to mechanical pressure exerted by tactical equipment (4). Furthermore, restrictive protective systems may reduce joint mobility and impair kinesthetic awareness, compromising movement precision and spatial judgment during operational tasks (14).

From an operational perspective, this perceptual distortion may be highly relevant. Underestimation of body dimensions may impair obstacle negotiation, movement efficiency, concealment strategies, and cover utilization during high-risk incidents. Previous studies involving firefighters and military personnel demonstrated that inaccurate spatial judgment while wearing PPE may increase collision risk and compromise tactical performance (15).

The present study has limitations that should be acknowledged, including its the design a within-subject, two-condition (PTU vs. OU) quasi-experimental comparison, relatively small sample size, and restriction to male police officers from a specialized tactical unit, which limits generalizability. Additionally, objective biomechanical and neuromuscular assessments were not performed. Future investigations should evaluate longitudinal adaptations to tactical equipment and incorporate advanced biomechanical analyses, wearable sensors, neurophysiological approaches and influence of BMI and lean and fat body mass to better understand the interaction between occupational load and perceptual-motor responses.

In conclusion, operational uniforms and PPE substantially increased musculoskeletal discomfort and promoted underestimation of body dimensions in special operations police officers. These findings reinforce the importance of tactical ergonomics, occupational health surveillance, and equipment optimization strategies aimed at reducing biomechanical overload and improving operational safety and performance. Furthermore, musculoskeletal injuries associated with operational equipment use may negatively affect occupational functionality and quality of life among police officers (21).

## Data Availability

The raw data supporting the conclusions of this article will be made available by the authors under reasonable request.

## References

[1] Schlenke J Nazzal Y Dogru F Holzgreve F Golbach R Karrasavidis I . Prevalence of musculoskeletal disorders among police officers from an organizational unit of a German federal state police force. J Occup Med Toxicol. (2026) 21:17. doi: 10.1186/s12995-026-00511-x 41998685 PMC13097821

[2] Manzolli SG Pinheiro MA Vieira LA Oliveira GS Tinti JC Fortes Junior PFC . Evaluation of lifestyle, physical activity level, and musculoskeletal discomfort of military police officers from the mounted police regiment. Fisioter Bras. (2024) 25:1865–82. doi: 10.62827/fb.v25i6.1038

[3] Marins EF Andrade LS Peixoto MB Silva MC. Frequency of musculoskeletal symptoms among police officers: systematic review. Br J Pain. (2020) 3:164–9. doi: 10.5935/2595-0118.20200034

[4] Knapik JJ Reynolds KL Harman E. Soldier load carriage: historical, physiological, biomechanical, and medical aspects. Mil Med. (2004) 169:45–56. doi: 10.7205/MILMED.169.1.45 14964502

[5] Blacker SD Carter JM Wilkinson DM Richmond VL Rayson MP Peattie M. Physiological responses of police officers during job simulations wearing chemical, biological, radiological and nuclear personal protective equipment. Ergonomics. (2013) 56:137–47. doi: 10.1080/00140139.2012.734335 23140326

[6] Frykman PN Harman EA Pandorf CE. Correlates of obstacle course performance among female soldiers carrying two different loads. Soldier Mobility. (2001) 9:1–9.

[7] Lewinski WJ Dysterheft JL Dicks ND Pettitt RW. The influence of officer equipment and protection on short sprinting performance. Appl Ergon. (2015) 47:65–71. doi: 10.1016/j.apergo.2014.08.017 25479975

[8] O'Neal EK Hornsby JH Kelleran KJ. High-intensity tasks with external load in military applications: a review. Mil Med. (2014) 179:950–4. doi: 10.7205/MILMED-D-14-00079 25181710

[9] Pryor RR Colburn D Crill MT Hostler DP Suyama J. Fitness characteristics of a suburban special weapons and tactics team. J Strength Cond Res. (2012) 26:752–7. doi: 10.1519/JSC.0b013e318225f177 22289693

[10] Ferraz AF Viana MV Rica RL Bocalini DS Battazza RA Miranda MLJ . Effects of physical activity on cardiometabolic parameters in police officers: a systematic review. Conscientiae Saúde. (2018) 17:356–70. doi: 10.5585/conssaude.v17n3.10283

[11] Canzoneri E Ubaldi S Rastelli V Finisguerra A Bassolino M Serino A. Tool-use reshapes the boundaries of body and peripersonal space representations. Exp Brain Res. (2013) 228:25–42. doi: 10.1007/s00221-013-3532-2 23640106

[12] Cardinali L Holmes NP. Body schema and tool use: current advances in cognitive neuroscience. Neurosci Biobehav Rev. (2023) 152:105316.

[13] Marins EF Cabistany L Bartel C Dawes J Del Vecchio FB. Effects of personal protective equipment on the performance of Federal Highway Policemen in physical fitness tests. J Strength Cond Res. (2020) 34:11–9. doi: 10.1519/JSC.0000000000003201 31268989

[14] Huck J. Protective clothing systems: a technique for evaluating restriction of wearer mobility. Appl Ergon. (1988) 19:185–90. doi: 10.1016/0003-6870(88)90136-6 15676659

[15] Petrucci MN Horn GP Rosengren KS Hsiao-Wecksler ET. Inaccuracy of affordance judgments for firefighters wearing personal protective equipment. Ecol Psychol. (2016) 28:108–26. doi: 10.1080/10407413.2016.1163987

[16] Rica RL Miranda JM Machado AF Evangelista AL Teixeira CLS Gama EF . Body-image and-size perception after a single session of HIIT body work in healthy adult men. Motricidade. (2018) 14:66–73. doi: 10.6063/motricidade.14914

[17] Ferraz AF Andrade EL Viana MV Rica RL Bocalini DS Figueira Júnior A. Physical activity level and sedentary behavior of military police staff. Rev Bras Med Esporte. (2020) 26:117–21. doi: 10.1590/1517-869220202602208923

[18] Holzgreve F Maltry L Lampe J Schmidt H Bader A Groneberg DA . Effects of occupational load carriage on postural stability and movement performance in tactical populations: implications for operational safety and occupational health. Front Public Health. (2026) 14:1489217.

[19] Neto AT Faleiro TB Moreira FD Jambeiro JS Schulz RS. Low back pain in the military police activity: analysis of prevalence, job impact and indirect cost. Rev Baiana Saude Publica. (2013) 37:365–74. doi: 10.22278/2318-2660.2013.v37.n2.a336

[20] Santos MMA Souza EL Barroso BIL. Analysis of the perception of state police officers regarding the comfort of bulletproof vests. Fisioter Pesqui. (2017) 24:157–62. doi: 10.1590/1809-2950/16629324022017

[21] Lima AG Santos JC. Physiotherapeutic intervention in musculoskeletal injuries in police officers caused by use of war materials: a literature review. Rev Cient Fac Educ Meio Ambiente. (2019) 10:178–82. doi: 10.31072/rcf.v10iedesp.625

